# Hepatitis C screening in hospitals: find the missing patients

**DOI:** 10.1186/s12985-019-1157-1

**Published:** 2019-04-16

**Authors:** Lili Liu, Hongqin Xu, Yue Hu, Jia Shang, Jianning Jiang, Lei Yu, Caiyan Zhao, Dazhi Zhang, Xinxin Zhang, Junfeng Li, Wei Li, Yanan Wu, Diefei Hu, Xiaofang Wang, Qian Zhao, Qiongfang Zhang, Wenqiang Luo, Jia Chen, Donghua Zhang, Wei Zhou, Junqi Niu

**Affiliations:** 1 Department of Hepatology, The First Hospital of Jilin University, Changchun, Jilin 130021 China; 2 Department of Phase I Clinical trial Center, The First Hospital of Jilin University, Changchun, Jilin 130021 China; 3 Department of Infectious Disease, Henan Provincial People’s Hospital, Zhengzhou, Henan 450003 China; 4 Department of Infectious Disease, The First Affiliated Hospital of Guangxi Medical University, Nanning, Guangxi 530021 China; 5 Department of Infectious Disease, The Fourth Hospital of Harbin Medical University, Harbin, Heilongjiang 150001 China; 6 Department of Infectious Disease, The Third Affiliated Hospital of Hebei Medical University, Shijiazhuang, Hebei 050051 China; 7 Department of Infectious Diseases, Institute of Viral Hepatitis, The Second Affiliated Hospital of Chongqing Medical University, Chongqing, 400010 China; 8 Department of Infectious Diseases, Ruijin Hospital, Shanghai Jiaotong University School of Medicine, Shanghai, 200025 China; 9 Department of Infectious Diseases, The First Hospital of Lanzhou University, Lanzhou, Gansu 730000 China; 10 Department of Neurosurgery, Second Affiliated Hospital, School of Medicine, Zhejiang University, Hangzhou, Zhejiang 31000 China

**Keywords:** Hepatitis C virus, Screening, Hospital, Positive rate

## Abstract

**Background:**

Hepatitis C virus (HCV) infection is one of the leading causes of liver cancer, creating enormous economic and social burdens. The Chinese government recommends routine screening of inpatients for HCV before invasive procedures to prevent iatric infections. However, the diagnosis and treatment rates for HCV remain low. The aim of this study was to use available routine screening data to understand the HCV screening of inpatients in different regions of China.

**Methods:**

Inpatient information and HCV screening results were collected from January 2016 to December 2016 at eight tertiary hospitals in different regions of China to compare the HCV-positivity of hospitalized patients among different regions and age groups.

**Results:**

The HCV screening rate of inpatients was more than 50%. A total of 467,008 inpatients were enrolled in the study (51.20% were male), and the HCV antibody (anti-HCV) -positive rate was 0.88% (95% confidence interval [CI], 0.85–0.91%) among the total population. This rate was significantly higher among all males compared with all females (0.91% vs 0.85%). Moreover, the HCV antibody-positive rate increased with age and was highest for the 60–64-year age group. Notably, 90.14% (3722/4129) of the anti-HCV seropositive patients were 40 years of age or older. HCV screening for people over 40 years old is recommended.

**Conclusions:**

This study highlights the key role of routine examination for HCV infection in hospitalized patients. Full use of inpatient screening results to manage HCV antibody-positive patients and a screening strategy targeting inpatients 40 years and older were found to be low-cost and effective, which will help to find the missing millions of yet unaware patients and also accelerate the elimination of HCV in China.

## Background

Hepatitis C virus (HCV) poses a serious public health problem due to its global prevalence. Acute HCV infection is often neglected because it manifests as only mild discomfort or can be asymptomatic, with gradual progression to severe liver disease, which ultimately lead to death. The absolute burden of viral hepatitis and related deaths increased tremendously between 1990 and 2013 [1]. Moreover, the HCV-related disease burden continues to increase as the infected population advances to late-stage liver disease [2]. A modeling study has indicated that improving the diagnosis and treatment rates of hepatitis C infection has a greater benefit on reducing disease burden than improving the efficacy of treatment [3]. Thus, the primary barrier that must be overcome to achieve HCV eradication is improving the rate of detection of infected patients.

In 2002, Japan’s government started a five-years national program to screen for HCV and HBV infection among people over 40 years of age [4]. In August 2012, the US Centers for Disease Control and Prevention recommended one-time screening for people born between 1945 and 1965 (i.e., the baby boomer cohort), regardless of other risk factors, and they have policies targeting at-risk populations [5]. In 2016, the World Health Organization (WHO) approved a global health sector strategy to eliminate hepatitis infection, aiming to reduce new cases of chronic hepatitis C and HCV-related deaths and to treat 80% of eligible chronic hepatitis C patients by 2030 [6, 7]. In 2018, the World Hepatitis Alliance (WHA) launched a global campaign entitled “Find the Missing Millions”, highlighting the importance of screening and linkage to care.

Due to its large population, a major portion of the global infectious disease burden arises in China. The most recent study showed that China has the largest number of hepatitis C patients among all countries, with approximately 9.8 million infected individuals [8, 9]. Unfortunately, it is estimated that less than 3% of those infected with HCV have been diagnosed, and by 2012, only half had been treated in China [10]. A large number of infected patients have not been discovered and treated. Thus, there is a huge gap in diagnosing infection among hepatitis C antibody-positive patients, as well as in meeting the 2030 treatment target.

At present in China, the task of greatest priority is improving the screening system in order to identify the infected population as soon as possible. At the same time, infected patients need to be linked with subsequent care after screening results are available. However, the implementation of universal screening for the whole population would require immense resources and would not be cost-effective. All hospitals in China conduct blood-borne virus screening for inpatients (particularly those who will undergo invasive examination, surgery and transfusion) to prevent iatrogenic infections [11], but further diagnosis and treatment are still lacking. If these screening results can be used to analyze the prevalence of HCV among the screened population and achieve early detection and treatment of patients, it would save a large amount of additional medical resources for screening.

Herein, we present the first study of HCV infection among hospitalized patients in different regions of China. It will provide an easy way to monitor the status of HCV infection in China. At the same time, our results provide valuable information for planning HCV screening and management strategies as well as for the rational allocation of resources.

## Materials and methods

### Study design and patients

We conducted a retrospective study of patients in eight hospitals located across seven different regions of China (North, Northeast, Northwest, East, Middle, South and Southwest). The participating hospitals were all large comprehensive tertiary hospitals in the provincial capital cities of all regions. The annual total number of inpatients is more than 850,000, and the sources of inpatients are widely distributed, including urban and rural patients in different regions. All hospitals contain dozens of departments, such as an emergency department, respiratory department, cardiovascular department, pediatrics department, obstetrics and gynecology department, traumatology department, burns surgery, ophthalmology department, otorhinolaryngology department, etc.

All patients admitted to these eight hospitals for inpatient treatment between January 2016 and December 2016 who did not refuse screening were included in this study, with the exception of inpatients treated in the liver disease departments of the hospitals as well as patients with incomplete data. The included patients were treated in widely distributed departments of the hospital. Data from repeat hospitalizations were only included for the first time. Data on socio-demographic characteristics were collected from electronic case records, and the results of HCV antibody testing were recorded for all patients. All patients were grouped into the respective age categories (i.e., 0 years, 1–4 years, 5–9 years, and so on).

In the clinical laboratories of hospitals in Changchun, Lanzhou, Shijiazhuang, Shanghai, and Guangxi, anti-HCV detection was performed by chemiluminescence immunoassay (CLIA) using an analyzer (Abbott, Architect i2000SR; Roche, Cobas 601; Vitro, Vitros eci/5600), whereas the detection of HCV antibody in the other three centers was performed by enzyme immunoassay (EIA) (Intec Diagnostic Kit and KHB Diagnostic Kit) on a TECAN analyzer (Freedom EVOlyzer, Männedorf, Switzerland). All the reagents were approved by the State Food and Drug Administration of China, and assays were carried out according to the manufacturers’ instructions. For the weak positive samples detected by EIA, CLIA was further applied as retesting. The result of CLIA were taken as the final result. All results of low antibody reactivity (i.e., equivocal) were considered negative in this study. Low antibody reactivity was defined by each laboratory according to the threshold signal-to-cut-off (S/CO) ratio for the test reagent.

This study was conducted in accordance with the Declaration of Helsinki, and the data collection was approved by the Institutional Review Boards of all Hospitals.

### Statistical analysis

The data were separated by age group and sex groups due to the complex sampling design of the study as well as according to response and analyzed using SPSS (SPSS, Inc., Chicago, IL, USA). Prevalence rates and constituent ratios as well as their 95% confidence intervals (CIs) for multi-morbidity were calculated. Differences in qualitative data were analyzed using the Chi-squared test. All *P* values are based on two-tailed tests, and *P* < 0.05 was considered to be statistically significant.

## Results

### Demographic characteristics of the study population

**Fig. 1:**
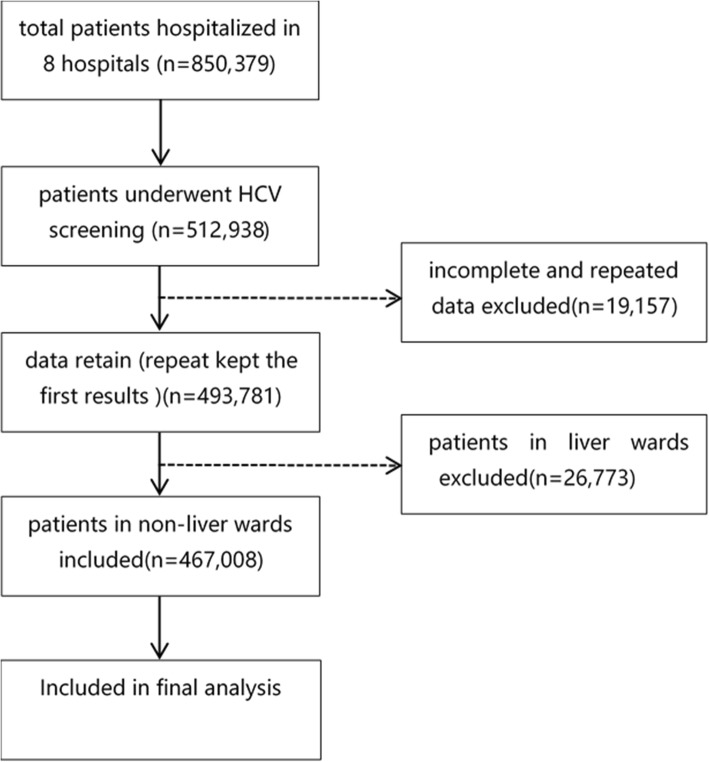
Data selection process

**Fig. 2:**
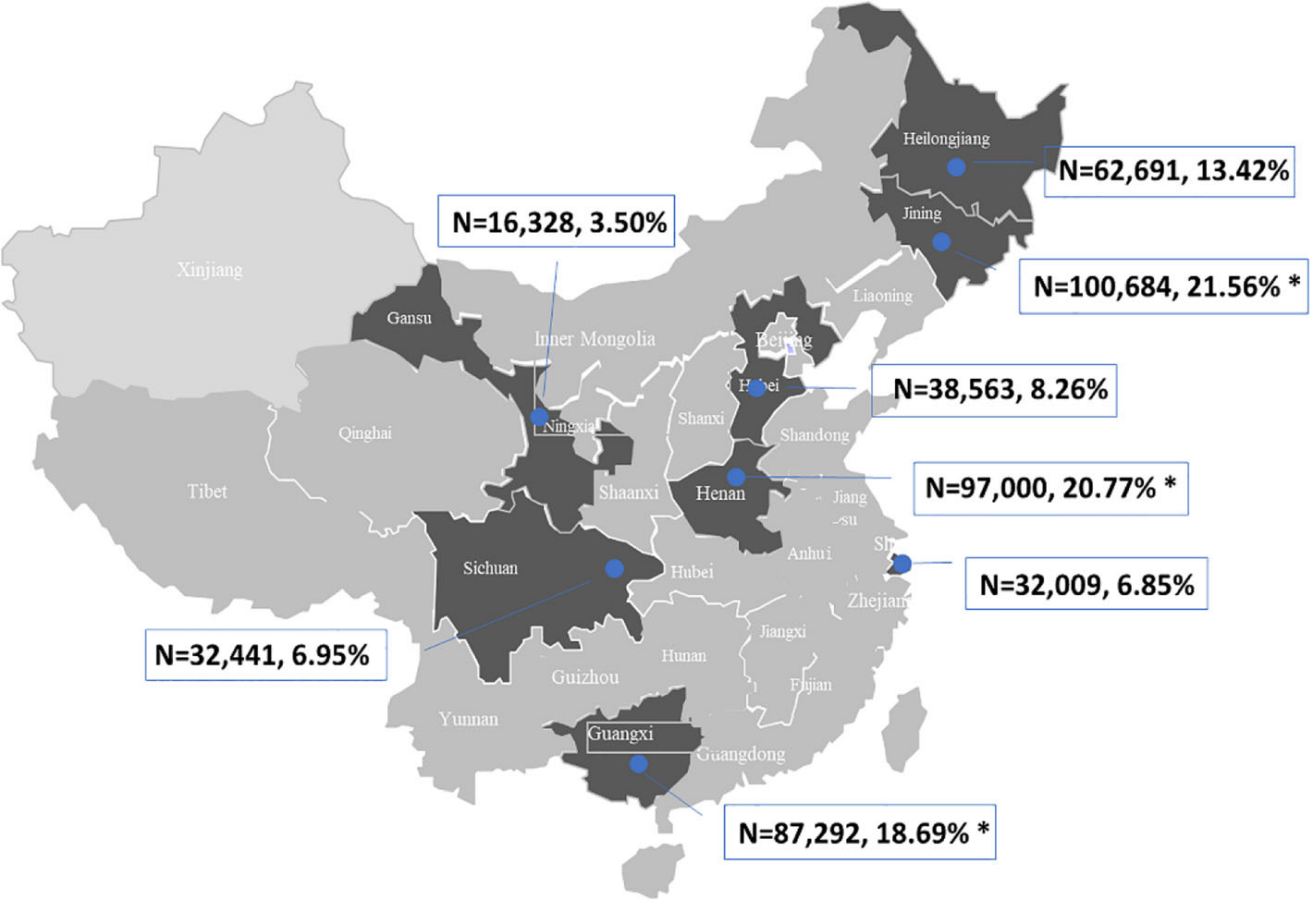
Distribution of participating hospitals across China. The provinces in which the participating hospitals are located are shaded, and the number of patients included in each hospital is shown with the corresponding percentage among the total study population. Asterisks denote the three hospitals with the largest numbers of included patients

Across the eight hospitals, 850,379 patients were hospitalized in 2016. A total of 467,008 patients treated in departments other than a liver disease department were finally included in the study (Fig. 1). The First Hospital of Jilin University in Changchun City, Jilin Province, the Henan Provincial People’s Hospital in Zhengzhou City, Henan Province, and the First Hospital of Guangxi Medical University in Nanning City, Guangxi Province accounted for the largest percentages of patients and are located in the northern, central and southern parts of China, respectively. The distribution of hospitals and the number of patients in each hospital are shown in Fig. 2. The study population included 239,103 (51.20%) male patients and 227,905 (48.80%) female patients. Patients were grouped by age. The 60–64-year group had the largest proportion of patients, and the 10–14-year group had the smallest proportion of patients (Table 1).

**Table 1:** Demographic characteristics of the included patients

	Number of patients	Proportion (%)
Sex		
Male	239,103	51.20
Female	227,905	48.80
Age (years)		
0	5598	1.20
1–4	13,392	2.87
5–9	9885	2.12
10–14	6698	1.43
15–19	8349	1.79
20–24	12,671	2.71
25–29	27,492	5.89
30–34	27,899	5.97
35–39	25,245	5.41
40–44	30,475	6.52
45–49	40,514	8.68
50–54	51,079	10.94
55–59	43,710	9.36
60–64^a^	53,312	11.41
65–69	40,889	8.75
70–74	27,823	5.96
> 75	41,977	8.99
Total	467,008	100

^a^The largest proportion of patients was aged 60–64 years

### Comparison of HCV antibody positivity rates in males and females of different age groups

**Fig. 3:**
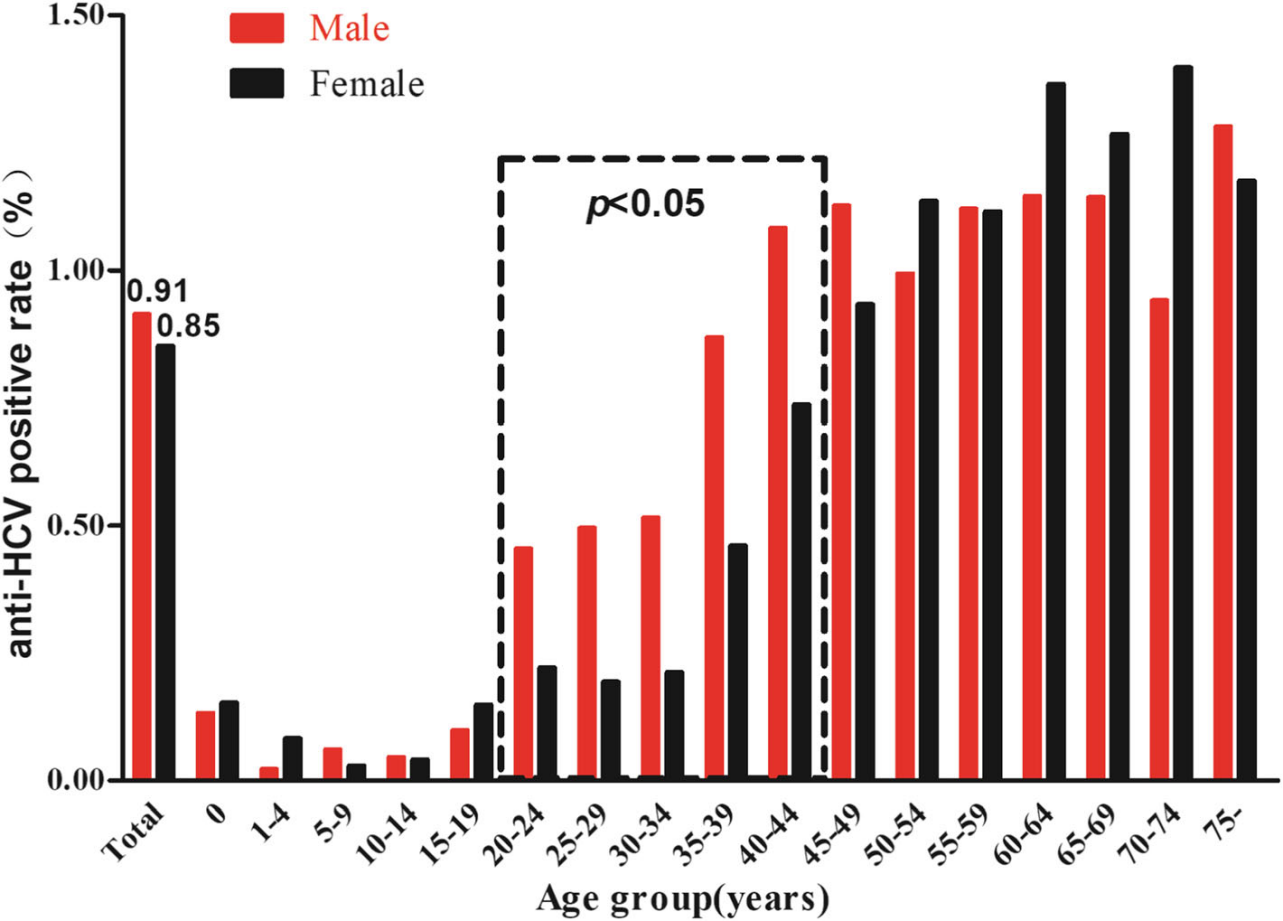
Comparison of HCV antibody-positive rates between males and females in different age groups. The overall anti-HCV antibody-positive rate in males and females are shown on the left. The HCV antibody-positive rates were less than 0.2% in all groups from 0 to 19 years of age, and the HCV antibody-positive rates were higher in men than in women in all age groups from 20 to 44 years (denoted by the dotted line)

The total number of HCV antibody-positive patients was 4129, for an HCV antibody- positive rate of 0.88% (95% CI, 0.85–0.91%). The HCV-positive rate in males was higher than that in females (0.91% vs 0.85%, *P <* 0.05). According to age group, the HCV antibody-positive rate gradually increased with age. Specifically, the HCV antibody-positive rate was lower than 0.2% in all groups less than 19 years, with no significant difference between males and females. In the age groups from 20 to 44 years, the HCV antibody-positive rate was significantly higher in men than in women. In men, the rate peaked in the 75–79-years age group at 1.28%, and the highest rate was observed in the 70–74-years group at 1.40% in women. Overall, 90.14% (3722/4129) of the anti-HCV seropositive patients were 40 years of age or older (Fig. 3).

### HCV antibody positivity in different regions of China

The prevalence of HCV infection varied among the different regions of China. Overall, the southern regions (including Chongqing and Nanning) had lower rates than the northern regions (including hospitals in Shijiazhuang in the north, Changchun and Harbin in the northeast, and Lanzhou in the northwest; 0.52% vs 1.1%). In the northern regions, Lanzhou had the highest positive rate for HCV antibody (1.75, 95%CI, 1.55–1.95%), and Shijiazhuang had the lowest positive rate (0.67, 95%CI, 0.59–0.75%). Among the eight regions, Nanning had the lowest positive rate for HCV antibody of only 0.47% (95%CI, 0.42–0.52%; Table 2).

**Table 2:** The positive rate of HCV antibody in each hospital

Hospital regions	Number of patients	Number of positive patients	Positive rate%	95%CI
Harbin	62,691	485	0.77	0.70–0.84
Changchun	100,684	1378	1.37	1.30–1.44
Lanzhou	16,328	286	1.75	1.55–1.95
Shijiazhuang	38,563	257	0.67	0.59–0.75
Zhengzhou	97,000	806	0.83	0.77–0.89
Shanghai	32,009	297	0.93	0.82–1.04
Chongqing	32,441	210	0.65	0.56–0.74
Nanning	87,292	410	0.47	0.42–0.52
Total	467,008	4129	0.88	0.85–0.91

**Fig. 4:**
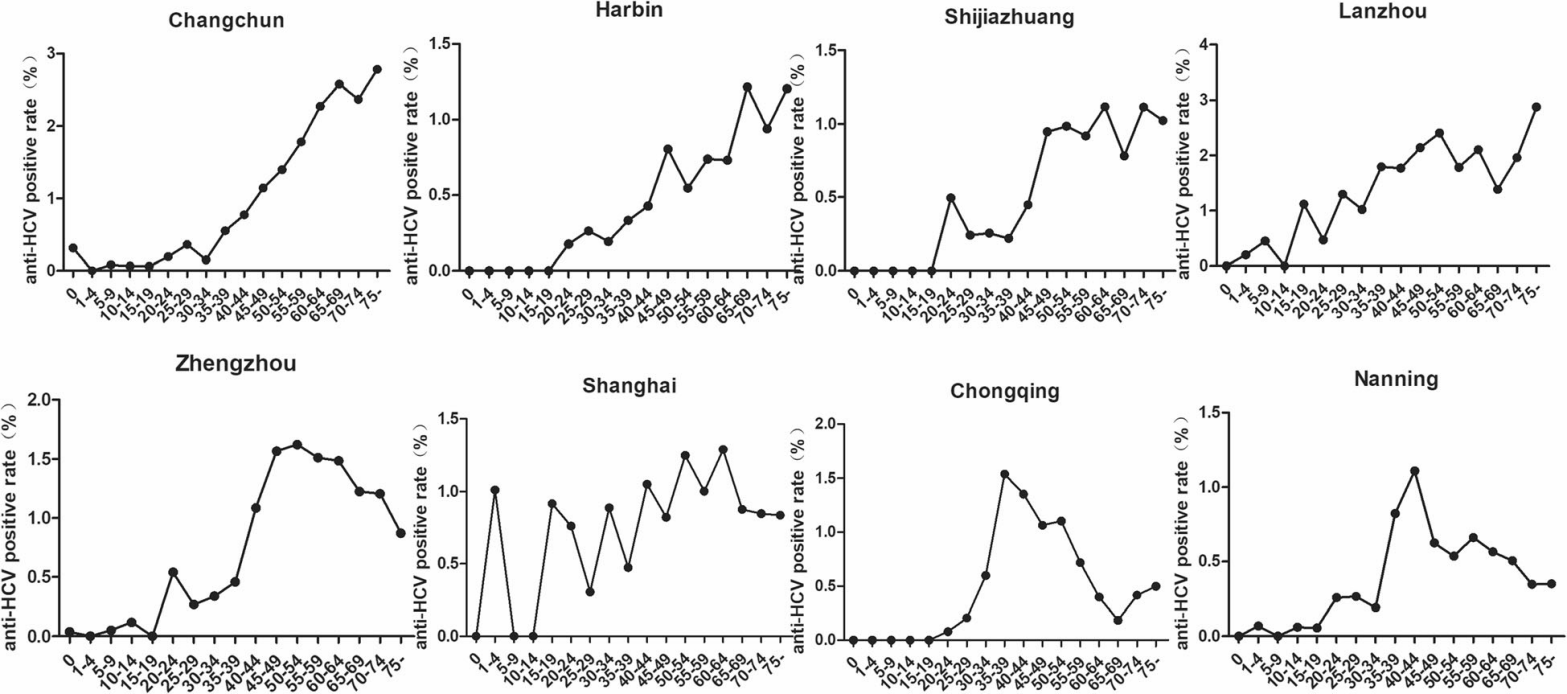
HCV antibody-positive rates in different regions according to age. The hospitals represented by the four graphs on top are located in four cities in the northern part of China, and those represented by the four graphs on bottom are located in four cities in the central, eastern, and southern parts of China

The HCV antibody-positive rate increased with age in the northern regions. However, in the central, eastern, and southern regions (including Zhengzhou, Shanghai, Chongqing, and Nanning), the positive rates were highest in middle-aged patients, with peak rates in the range of 40–64 years of age. In all regions except Shanghai and Lanzhou, the rate of anti-HCV positivity was lowest in the age groups from 0 to 19 years (Fig. 4).

### National estimate of HCV antibody detection

The number of inpatients across the eight institutions was about 850,000, and 467,008 inpatients receiving treatment in departments other than a liver disease department were screened in 2016. The HCV screening rate was more than 50% (after removal of repeat hospitalizations). According to the National Health Statistics Yearbook data, 227 million individuals received inpatient care in China in 2016 [12]. Based on the overall HCV antibody-positivity rate, the number of inpatients who were positive for HCV antibodies was estimated at 2 million. If the HCV screening rates of other hospitals in China were consistent with 50% of our results, approximately 1 million cases (in non-hepatic departments) were found in the routine examination of hospitalized patients in 2016.

## Discussion

The results of the present study reveal several important features of HCV infection in Chinese inpatients. The rate of HCV screening in the departments of non-liver diseases was more than 50%. The overall HCV infection rate among hospitalized patients in China was 0.88%. The HCV antibody-positivity rate gradually increased with age and was higher in the northern regions of China than in the southern regions.

The overall positive rate was similar to rates of 0.7–1% reported for hospitalized patients in Turkey [13]. Furthermore, our result is consistent with a global study of 2017 conducted by the Polaris Observatory HCV Collaborators, which reported an estimated prevalence of viraemic HCV of 0.7% (95% uncertainty interval 0.5–0.8%) in China [8]. Therefore, we conclude that it is feasible to assess the overall HCV antibody status in China based on hospital inpatient screening results. Moreover, a large population of infected people was identified through screening, providing a basis for the development of strategies for diagnosis, evaluation, treatment, and follow-up.

HCV infection occurred at a higher rate in males than in females, especially in the 20–44-year age group, and other studies have reported conflicting results regarding this topic [14–16]. We hypothesize that men may have more chances to be infected by HCV through unhealthy lifestyles or behaviors, such as male homosexuality, sharing of equipment used for drug injection, etc [17]. The overall HCV antibody-positive rate gradually increased with age. This is consistent with the findings of a previous cross-sectional study in northeastern China [18]. Since the widespread implementation of measures to improve medical safety in China in the early 1990s, the number of cases of hepatitis C transmitted through unsafe medical practices has decreased significantly. This may partly explain why the anti-HCV positive rate was lowest among inpatients less than 20 years old, with rates all below 0.2% in the specific age groups. Moreover, people aged 0–19 years of age were all born after 1993, the year when China implemented the screening of blood donors for HCV antibody [19]. This measure reduced the risk of infection from blood transfusions. Among the included subgroups, HCV antibody positivity was slightly higher in 0-year-old patients, which may be related to passive acquisition of antibodies from HCV-positive mothers. The rate of vertical transmission from infected mothers to infants was reported to be about 6% [20]. Patients 40 years of age and older had the highest rates of HCV positivity, and two factors may be involved: 1) these patients could have participated in a series of dangerous behaviors, such as, blood transfusion, tattoos, and unclean injections before HCV was discovered, leading to an increased risk of infection; 2) In the higher age group, the less educated people accounted for a larger proportion, and there were fewer opportunities to learn about HCV through books, the internet, or other methods. The HCV antibody-positive rate decreases among those born at later dates, which fully demonstrates the effectiveness of screening for blood donation and the implementation of management policies for HCV prevention.

Notably, one age group (≥40 years) accounted for the majority of infected persons (> 90%). Consistently, a study in Hebei province found that 98.2% of participants who were positive for HCV RNA were aged > 40 years [21]. This finding shows that the cumulative infection rate and morbidity rate in this portion of the population during the decades were higher than those in younger people, which is consistent with the increase in the prevalence of HCV positivity in the high-age group obtained via the annual reported incidence [22]. Considering that this age group includes many working individuals, HCV-related disease will affect productivity among the population and bring huge economic losses and a great social burden to the country. In addition, people over 40 years old with HCV infection are at high risk for HCC. Finding these patients as early as possible can increase the screening of high-risk groups, so as to achieve early detection of HCC. A similar screening strategy launched in Japan, which screened people over the age of 40 for hepatitis B and C, achieved early detection in more than 60% of HCC patients [23]. Several studies of economic assessment have demonstrated the cost-effectiveness of HCV screening primarily in populations with elevated prevalence [24, 25]. Thus, it is imperative to strengthen HCV screening for people 40 years of age and older and to improve HCV-related HCC surveillance in this age group. It would be a low-cost and effective strategy of screening compared to universal screening.

The positive rate of HCV antibody in the northern regions was higher than that in the southern regions, which was consistent with the previous epidemiological findings [26]. We speculate that the wide spread of parenteral caffeinum natrio-benzoicum and sharing of syringes in some northern regions in the 1980s resulted in a regional epidemic of HCV [27]. This could also explain why the peak HCV positive rates in the northern regions were mostly in the middle and old age groups. A different trend was observed in other regions where rates were highest among middle-age inpatients. This may be related to the relatively developed economy and dense population of the central and southern parts of the country, which may lead to more injected opioid use and promiscuous sexual behaviors. A study conducted in Southwest China reported similar results, with the highest rate of HCV positivity in the 45–54-year age group [16]. This result suggests that even within the same country, government programs should be implemented according to the epidemic characteristics of different regions, and health education for young and middle-aged people should be strengthened.

As a benefit of the national policy implemented by the government, the number of HCV-positive patients routinely examined in hospitals is large, but further examinations and treatments are ignored due to lack of knowledge about hepatitis C prevention and treatment in other departments. This problem is the same in other countries, with a study of the United States revealing that less than 50% of anti-HCV positive persons had received confirmatory HCV RNA testing, and among those with a confirmed diagnosis of HCV infection, only a minority (approximately 16%) were prescribed treatment [28]. In addition, there is no obvious clinical symptom in the early stage of hepatitis C. Millions of patients are unaware of their infections, which leads to a gap between infection and effective treatment. The United States has already made some attempts to integrate an alert into electronic health record systems and has found that HCV screening increased the screening rate by 5-fold and improved the HCV diagnosis and linkage to care [29]. Based on our results, the screening rate of HCV of inpatients in Chinese hospitals is high, and a large number of people are screened every year. If we make full use of the available resources to identify the millions of not yet diagnosed patients and facilitate treatment of infected patients, it is expected that we will accelerate the pace of elimination of HCV.

Our study does include several limitations as follows. First, this study was a descriptive analysis using hospital screening data rather than a seroprevalence study. The results represent HCV detection in the hospitalized population. Second, due to the retrospective nature of our study, we focused on assessing HCV antibody-positive results in hospitalized patients and did not perform further HCV RNA quantification. Health education for HCV-antibody positive patients and referral to a specialist for further diagnosis will be our next focus. Finally, the HCV antibody detection methods and instruments of 8 hospitals were not completely consistent. In order to reduce the false positive rate, we considered the weakly positive results to be negative, and thus, our anti-HCV positive rate may be slightly lower than the real level. Nevertheless, the number of HCV antibody-positive patients found through routine screening is still very large.

To our knowledge, this is the first multi-center study on the screening of inpatients in China. The sample size is large, and the patient sources are widely distributed. Thus, this study can accurately reflect the real world situation and describe the current prevalence of HCV in China. Based on the current research, it can be speculated that the full use of existing medical resources will facilitate the completion of screening of the majority of the HCV-infected population in China.

## Conclusions

The present study shows that the rate of positive anti-HCV detection decreases with decreasing age among inpatients, which demonstrates the positive effects of related government efforts. Furthermore, this study highlights the key role of routine examination in hospitalized patients. Full use of inpatient screening supports the management of HCV antibody-positive patients, and a screening strategy targeting inpatients 40 years and older was shown to be low-cost and effective. Thus, such programs will help to find the missing millions of patients who remain unaware and/or undiagnosed and also accelerate the elimination of HCV in China.

## Abbreviations

anti-HCV: HCV antibody
CI: confidence interval
CLIA: chemiluminescence immunoassay
EIA: enzyme immunoassay
HCC: hepatocellular carcinoma
HCV: hepatitis C virus
S/CO: signal-to-cut-off
WHA: World Hepatitis Alliance
WHO: World Health Organization

## References

[1] Stanaway JD, Flaxman AD, Naghavi M, Fitzmaurice C, Vos T, Abubakar I, Abu-Raddad LJ, Assadi R, Bhala N, Cowie B (2016). The global burden of viral hepatitis from 1990 to 2013: findings from the global burden of disease study 2013. Lancet.

[2] Gane E, Kershenobich D, Seguin-Devaux C, Kristian P, Aho I, Dalgard O, Shestakova I, Nymadawa P, Blach S, Acharya S (2015). Strategies to manage hepatitis C virus (HCV) infection disease burden - volume 2. J Viral Hepat.

[3] Volk ML, Tocco R, Saini S, Lok AS (2009). Public health impact of antiviral therapy for hepatitis C in the United States. Hepatology.

[4] Tanaka J, Yoshizawa H (2004). A national project for the management of viral hepatitis toward prevention of hepatocellular carcinoma in Japan. Gan To Kagaku Ryoho.

[5] Smith BD, Morgan RL, Beckett GA, Falck-Ytter Y, Holtzman D, Teo CG, Jewett A, Baack B, Rein DB, Patel N (2012). Recommendations for the identification of chronic hepatitis C virus infection among persons born during 1945-1965. MMWR Recomm Rep.

[6] World Health Organization. Draft Global Health sector strategies viral hepatitis 2016–2021**.** 2016. Available at: https://www.who.int/hepatitis/strategy2016-2021/ghss-hep/en/ (Accessed Jun 2016); WHO.

[7] World Health Organization. Combating Hepatitis B and C to Reach Elimination by 2030. 2016. Available at: https://www.who.int/hepatitis/publications/hep-elimination-by-2030-brief/en/ (Accessed May 2016); WHO.

[8] Blach S, Zeuzem S, Manns M, Altraif I, Duberg A-S, Muljono DH, Waked I, Alavian SM, Lee M-H, Negro F (2017). Global prevalence and genotype distribution of hepatitis C virus infection in 2015: a modelling study. The Lancet Gastroenterology & Hepatology.

[9] Lavanchy D (2011). Evolving epidemiology of hepatitis C virus. Clin Microbiol Infect.

[10] Wei L, Lok AS (2014). Impact of new hepatitis C treatments in different regions of the world. Gastroenterology.

[11] National health commission of the People's Republic of China (2015). Screening and Management of Viral Hepatitis C. Inform Infect Dis.

[12] National Health Commission of the People's Republic of China (2017). The year book of health in the People’s republic of China.

[13] Turhanoglu M, Onur A, Bilman FB, Ayaydin Z, Aktar GS (2013). Eight-year seroprevalence of HBV, HCV and HIV in Diyarbakir training and research hospital. Int J Med Sci.

[14] Lu J, Zhou Y, Lin X, Jiang Y, Tian R, Zhang Y, Wu J, Zhang F, Zhang Y, Wang Y, Bi S (2009). General epidemiological parameters of viral hepatitis A, B, C, and E in six regions of China: a cross-sectional study in 2007. PLoS One.

[15] Kim DY, Kim IH, Jeong SH, Cho YK, Lee JH, Jin YJ, Lee D, Suh DJ, Han KH, Park NH (2013). A nationwide seroepidemiology of hepatitis C virus infection in South Korea. Liver Int.

[16] Zhou M, Li H, Ji Y, Ma Y, Hou F, Yuan P (2015). Hepatitis C virus infection in the general population: a large community-based study in Mianyang, West China. Biosci Trends.

[17] Degenhardt L, Peacock A, Colledge S, Leung J, Grebely J, Vickerman P, Stone J, Cunningham EB, Trickey A, Dumchev K (2017). Global prevalence of injecting drug use and sociodemographic characteristics and prevalence of HIV, HBV, and HCV in people who inject drugs: a multistage systematic review. Lancet Glob Health.

[18] Zhang Q, Qi W, Wang X, Zhang Y, Xu Y, Qin S, Zhao P, Guo H, Jiao J, Zhou C (2016). Epidemiology of hepatitis B and hepatitis C infections and benefits of programs for hepatitis prevention in northeastern China: a cross-sectional study. Clin Infect Dis.

[19] National Health Commission of the People’s Republic of China. Blood transfusion services and blood management. 1993. Available at: http://www.chinalawedu.com/falvfagui/fg22598/181602.shtml. Accessed Mar 1993.

[20] Benova L, Mohamoud YA, Calvert C, Abu-Raddad LJ (2014). Vertical transmission of hepatitis C virus: systematic review and meta-analysis. Clin Infect Dis.

[21] Xu CJ, Zhang CP, Luo BF, Liu LJ, Wang YZ, Wang XH, He QJ, Zhou SS, Guo WS, Wang JH (2015). Prevalence and characterization of hepatitis B and C virus infections in a needle-sharing population in northern China. BMC Public Health.

[22] Liu Z, Yang Q, Shi O, Ye W, Chen X, Zhang T (2018). The epidemiology of hepatitis B and hepatitis C infections in China from 2004 to 2014: an observational population-based study. J Viral Hepat.

[23] Omata M, Lesmana LA, Tateishi R, Chen PJ, Lin SM, Yoshida H, Kudo M, Lee JM, Choi BI, Poon RT (2010). Asian Pacific Association for the Study of the liver consensus recommendations on hepatocellular carcinoma. Hepatol Int.

[24] Coward S, Leggett L, Kaplan GG, Clement F (2016). Cost-effectiveness of screening for hepatitis C virus: a systematic review of economic evaluations. BMJ Open.

[25] Sroczynski G, Esteban E, Conrads-Frank A, Schwarzer R, Muhlberger N, Wright D, Zeuzem S, Siebert U (2009). Long-term effectiveness and cost-effectiveness of screening for hepatitis C virus infection. Eur J Pub Health.

[26] Chen Y, Li L, Cui F, Xing W, Wang L, Jia Z, Zhou M, Gong X, Wang F, Zheng H (2011). A sero-epidemiological study on hepatitis C in China. Chin J Epidemiol.

[27] Xu H, Yu G, Sun H, Lv J, Wang M, Kong F, Zhang M, Chi X, Wang X, Wu R (2015). Use of parenteral caffeinum natrio-benzoicum: an underestimated risk factor for HCV transmission in China. BMC Public Health.

[28] Yehia BR, Schranz AJ, Umscheid CA, Lo Re V (2014). The treatment cascade for chronic hepatitis C virus infection in the United States: a systematic review and meta-analysis. PLoS One.

[29] Konerman MA, Thomson M, Gray K, Moore M, Choxi H, Seif E, Lok ASF (2017). Impact of an electronic health record alert in primary care on increasing hepatitis c screening and curative treatment for baby boomers. Hepatology.

